# Cyclic Guanosine Monophosphate Modulates Locomotor Acceleration Induced by Nitric Oxide but not Serotonin in *Clione limacina* Central Pattern Generator Swim Interneurons

**DOI:** 10.1093/iob/obaa045

**Published:** 2021-01-24

**Authors:** Thomas J Pirtle, Richard A Satterlie

**Affiliations:** Department of Biology, The College of Idaho, 2112 Cleveland Blvd Caldwell, ID 83605, USA; Department of Biology and Marine Biology and Center for Marine Science, University of North Carolina Wilmington, 5600 Marvin K. Moss Road, Wilmington, NC 28409, USA

## Abstract

Both nitric oxide (NO) and serotonin (5HT) mediate swim acceleration in the marine mollusk, *Clione*  *limacina*. In this study, we examine the role that the second messenger, cyclic guanosine monophosphate (cGMP), plays in mediating NO and 5HT-induced swim acceleration. We observed that the application of an analog of cGMP or an activator of soluble guanylyl cyclase (sGC) increased fictive locomotor speed recorded from Pd-7 interneurons of the animal’s locomotor central pattern generator. Moreover, inhibition of sGC decreased fictive locomotor speed. These results suggest that basal levels of cGMP are important for slow swimming and that increased production of cGMP mediates swim acceleration in *Clione*. Because NO has its effect through cGMP signaling and because we show herein that cGMP produces cellular changes in *Clione* swim interneurons that are consistent with cellular changes produced by 5HT application, we hypothesize that both NO and 5HT function via a common signal transduction pathway that involves cGMP. Our results show that cGMP mediates NO-induced but not 5HT-induced swim acceleration in *Clione*.

## Introduction

Central pattern generators (CPGs) are networks of neurons that are capable of creating rhythmic movements (Delcomyn 1980; Marder and Bucher 2001; Clarac and Pearlstein 2007; Mullins et al. 2011). Studies of CPGs span the animal phyla and include nematodes (Karbowski et al. 2008), arthropods (Selverston 2005; Vierk et al. 2009, Marder 2010), annelids (Eisenhart et al. 2000; Kristan et al. 2005; Friesen 2010), mollusks (Satterlie 1985; Arshavsky et al. 1998; Straub et al. 2002; Thompson and Watson 2005; Arshavsky et al. 2010; Katz 2010), and chordates (Grillner and Wallén 2010; Horie et al. 2010). A common feature of many CPGs is that they must be pliant so as to transform an animal’s behavior in response to a large number of inevitable environmental conditions. The ability of CPGs to produce behavioral shifts requires neuromodulation that may consist of modification to network, synaptic, and cellular properties of CPG neurons (Dickinson 2006). Neuromodulation may occur through ionotropic receptors that have a direct effect on membrane potential or through signal transduction mechanisms, in which an extracellular neuromodulator binds to metabotropic receptors that are subsequently transduced into chemical signals, called second messengers, within the component neurons that comprise the CPG. Important second messengers include cyclic nucleotides (e.g., cyclic adenosine monophosphae [cAMP] and cyclic guanosine monophosphate [cGMP]), inositol triphosphate and diacylglycerol, and calcium ions (Worley et al. 1987; Kaczmarek and Levitan 1987a; Katz and Clemens 2001; Marder 2012).

Some examples of second messenger systems in modulating CPGs include the role of cGMP in inhibiting swimming in *Melibe* (Newcomb and Watson 2002), the role of cAMP in neuron C2 of the escape swim CPG of *Tritonia* (Clemens et al. 2007), and the role of cAMP in modifying motor frequency produced by CPG neurons comprising the stomatogastric ganglion of decapod crustaceans (Flamm et al. 1987; Hempel et al. 1996). Second messengers produce their effect in excitable cells by modifying the ionic conductance of excitable cell membranes. Hence, second messengers can affect the membrane potential, the duration and amplitude of action potentials, and the timing of action potentials (Kaczmarek and Levitan 1987b; Hille 1992). Here, we describe the role of cGMP in mediating changes in locomotor speed in the pteropod mollusk, *Clione*.

*Clione* is negatively buoyant and must maintain its position by an archetypal slow locomotor gait that involves the rhythmic dorsoventral movement of its wing-like parapodia. Swimming in *Clione* is typically slow and orients the long axis of the animal’s body perpendicular to the water surface. However, swimming speed may increase in response to stimuli applied to the tail during escape behavior or during hunting and feeding (Arshavsky et al. 1985a; Satterlie 1989). Previous experiments have shown that bath application of serotonin (5HT; 1–100 µM) to reduced preparations of *Clione* increased fictive swim frequency recorded from swim interneurons of the animal’s swim CPG (Panchin et al. 1996; Satterlie et al. 2000; Satterlie and Norekian 2001; Pirtle et al. 2010). Furthermore, 5HT also produces several cellular changes in swim interneurons that include baseline depolarization, reduction in action potential duration (spike narrowing), changes to postinhibitory rebound (PIR; increased amplitude of PIR and decreased latency to peak PIR), and enhanced sag potential amplitude (Satterlie 1991; Satterlie, et al. 2000; Pirtle and Satterlie 2007). In addition to 5HT, nitric oxide (NO) also increases fictive locomotor speed in *Clione* (Moroz et al. 2000). Here we show that cGMP produces many of the 5HT-induced cellular changes to *Clione* swim interneurons, and test the hypothesis that both 5HT and NO produce their effects through a cGMP signal transduction mechanism.

## Materials and methods

### Animals and animal preparation

*Clione limacina* was collected by dipping them off the breakwater at Friday Harbor Laboratories, Friday Harbor, Washington, USA during the months of May and June. Animals used in experiments were maintained in 1-gallon jars (filled with seawater filtered with bag filters) that were partly submerged in sea tables containing circulating seawater. Seawater in the jars was replaced twice daily to help maintain the health of the animals.

*Clione* used in experiments were selected with a length of 1.0–1.5 cm. Selected animals were anesthetized in a mixture of bag filtered seawater and refrigerated (4°C) isotonic (0.333 M) MgCl_2_. Anesthetized animals were pinned using cactus spines (*Opuntia sp*.) dorsal side up in Sylgard^®^ lined 3.3-cm diameter Petri dishes (Corning) and dissected into a reduced preparation consisting of parapodia (wings) and isolated ganglia (paired cerebral, pleural, pedal, and intestinal ganglia). Approximately 1 mg of protease Type XIV (Sigma-Aldrich, St Louis, MO, USA) was added directly over the dissected preparation and allowed to dissolve into the seawater bathing the preparation to digest the connective tissue sheath that surrounds the ganglia. An approximate measure of protease Type XIV was used because the action of protease Type XIV was visually monitored under a dissecting microscope until the connective tissue sheath was observed to separate from the ganglia. Type XIV protease was thoroughly washed out of the bath with 100 mL of bag filtered seawater following the chemical digestion of the connective tissue sheath. Dissection of *Clione* and Type XIV protease treatment were carried out at room temperature (20°C). However, after protease treatment, the preparation was transferred to a cooling stage (Dagan Corporation) to maintain the experimental temperature at 10 ± 1°C.

### Pharmacological agents

Several drugs were used in experiments and applied from stock solutions to the bathing seawater to test our hypothesis. Inhibitors of soluble guanylyl cyclase (sGC; 1H-[1,2,4]oxadiazolo[4,3-a]quinoxalin-1-one [ODQ]) and protein kinase G (2-bromo-3,4-dihydro-3-[3,5-O-[(R)-mercaptophosphinylidene]-β-d-ribofuranosyl]-6-phenyl-9H-imidazo[1,2-a]purin-9-one sodium salt [Rp-8-Br-PET-cGMP]) were allowed fifteen minutes to exert their effect prior to recording the drug’s effect. In some experiments, the combined effects of drugs were shown by the co-application of drugs. In these experiments, the effects of one drug were observed before applying the second drug to the bath. Washing the preparation with 20 mL of filtered seawater removed the drug or combination of drugs. Previous experiments had been conducted to determine the lowest concentration of each drug to yield a reliable change in fictive swim frequency and we follow the same protocols for making stock solutions of these chemicals used in this study (for 5HT and mianserin see Satterlie, et al. 2000; for sodium 2-(N, N-diethylamino)-diazenolate-2-oxide [DEANO], 8-Bromoguanosine cyclic 3′,5′-monophosphate sodium salt [8-Br-cGMP], and ODQ see Moroz et al. 2000; Walton et al. 2007; Walton and Pirtle 2009, for tetrodotoxin [TTX], atropine, and 6-Cyano-7-nitroquinoxaline-2,3-dione disodium [CNQX] see Pirtle and Satterlie 2007). Concentrations of chemicals not previously used with *Clione* were based upon concentrations reported in the literature for *Aplysia californica* (for L-NAME and 2-(4-carboxyphenyl)-4,4,5,5-tetramethylimidazoline-1-oxyl-3-oxide [PTIO], see Ye et al. 2010 and Miller et al. 2011). For Rp-8-Br-PET-cGMP, we used a concentration based upon the use of a similar PKG inhibitor, Rp-8-pCPT-cGMPS, used in *A. californica* (Lewin and Walters 1999).

The NO donor, DEANO, the soluble cyclic guanylyl cyclase activator (E)-1-(2,4-dihydroxyphenyl)-3-(4-hydroxyphenyl)-2-propen-1-one, 4,2′,4′-trihydroxychalcone (isoliquiritigenin), the NO scavenger, PTIO, and 3-[2-Aminoethyl]-5-hydroxyindole creatinine sulfate complex (5HT) were purchased from Sigma-Aldrich. DEANO was made in filtered seawater immediately before use and applied quickly to the bath to show the effects of NO on the *Clione* swim interneurons. The NO activated sGC inhibitor, ODQ, 8-Br-cGMP, Rp-8-Br-PET-cGMP, octahydro-12-(hydroxymethyl)-2-imino-5,9:7,10a-dimethano-10aH-[1,3]dioxocino[6,5-d]pyrimidine-4,7,10,11,12-pentol citrate (TTX), CNQX were purchase from Tocris R&D Systems, Minneapolis, MN, USA. To make the drug stock solution dissolve in seawater, ODQ and isoliquiritigenin were dissolved in dimethyl sulfoxide (DMSO; Sigma-Aldrich). The total DMSO concentration in the seawater bathing the preparation was 0.1%—a concentration of DMSO that had previously been shown not to affect recordings from *Clione* swim interneurons (Walton et al. 2007; Walton and Pirtle 2009). ODQ was co-applied in experiments with DEANO or 5HT, and in these experiments, ODQ was applied prior to applying the other drugs. Similarly, Rp-8-Br-PET-cGMP was co-applied with 5HT and in this experiment, Rp-8-Br-PET-cGMP was applied prior to applying 5HT. A constant volume of 3 mL was maintained in the recording dish and final concentrations of each chemical are provided in the results.

### Electrophysiological data collection, data analysis, and statistics

Electrophysiology involved the intracellular recording of electrical activity from Pd-7 swim interneurons of *C. limacina*. Pd-7 interneurons were identified based on the location of these neurons within the pedal ganglia and also their electrophysiology (Satterlie 1985; Arshavsky et al. 1985b). An Axon Instruments Axoclamp 2B or A-M Systems amplifiers were used to record electrical activity from Pd-7 interneurons. Intracellular electrodes were made from thin-wall borosilicate glass (World Precision Instruments product TW100F-4) using a Sutter Instruments P-97 Flaming/Brown electrode puller and filled with 2 M potassium acetate. Electrodes had a resistance between 20 and 30 MΩ. Data were recorded using Axon Instruments, Inc. Pclamp or Axoscope software and A/D converter on a laptop PC. PClamp software (when using the Axon Instruments Axoclamp 2B) was used for current injection protocols (Molecular Devices, LLC San Jose, CA, USA).

Measurements of fictive swim frequency recorded from Pd-7 interneurons of the *Clione* swim CPG involved dividing the number of cycles (typically 10) by the duration of time of those cycles. Statistical analysis was done using GraphPad Instat (GraphPad Software, La Jolla, CA, USA) and all graphs were created using Origin 2020 (OriginLab Corporation, Northampton, MA, USA). All data are reported as mean ± standard error. The Friedman test (nonparametric repeated measures analysis of variance [ANOVA]) was used to identify significant differences between group means. Either a paired two-tailed *t*-test or an unpaired two-tailed *t*-test with Welch correction was used when comparing two means. Significance is determined by a *P* ≤0.05 and indicated graphically by lower case letters.

## Results

### Both 5HT and NO independently increase swim locomotor speed

5HT increases fictive swim speed in *Clione* (Satterlie and Norekian 1995, 1996; Panchin et al. 1996; Satterlie et al. 2000), and the serotonergic neurons that regulate swim activity are also well known (Satterlie and Norekian 1995, 1996; Panchin et al. 1996). However, we demonstrate here that 5HT produces its effect independent of NO. 5HT (1 µM) induces fictive swim acceleration in the isolated *Clione* nervous system when co-applied to preparations previously exposed to the NO scavenger, PTIO (100 µM), and the NOS inhibitor, L-NAME (100 µM). The mean fictive swimming speed recorded in the presence of PTIO and L-NAME only (recorded 15 min after adding PTIO and L-NAME simultaneously) is 1.70 ± 0.112s^−1^. However, adding 5HT in the presence of previously added PTIO and L-NAME increased mean fictive swim speed to 2.90 ± 0.287s^−1^. Thus, the co-application of 5HT to preparations previously exposed to PTIO and L-NAME significantly increased the fictive swimming speed 71% (*N *=* *5; *P *=* *0.0055; Friedman test, nonparametric repeated measures ANOVA, with Dunn multiple comparison post-test).

Similarly, NO increases fictive swim speed in *Clione* (Moroz et al. 2000), and we demonstrate here that NO produces its effect independent of 5HT. The NO donor, DEANO (50 µM), induces fictive swim acceleration in the isolated *Clione* nervous system when co-applied to preparations previously exposed to the 5HT receptor antagonist, mianserin (10 µM). The mianserin added to seawater served as the control in these experiments because mianserin has been previously shown to block the effects of 5HT in *Clione* (Satterlie et al. 2000). The mean fictive swimming speed recorded in the presence of mianserin only (recorded 15 min after adding mianserin in seawater) is 1.52 ± 0.140s^−1^. However, adding DEANO increased fictive swim speed to 2.78 ± 0.380s^−1^ in the presence of mianserin. Thus, the co-application of NO to preparations already exposed to mianserin increased the fictive swimming speed 83% (*N *=* *6; *P *=* *0.0081; Friedman test, nonparametric repeated measures ANOVA, with Dunn multiple comparison post-test).

### Effects of cGMP on the clione swim locomotor rhythm

**Fig. 1:**
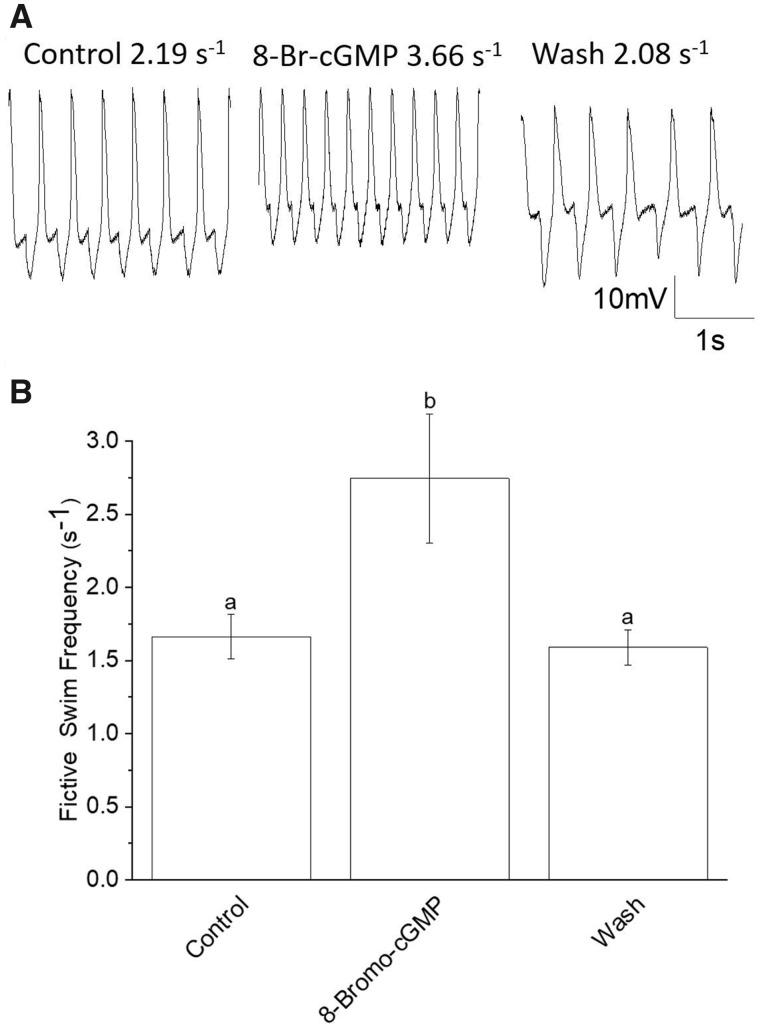
Effects of the cell membrane permeable cGMP analog, 8-Br-cGMP (200 μM), on fictive swim frequency recorded from *Clione* Pd-7 swim interneurons. (**A**) An example of the application of 200 μM 8-Br-cGMP. In this example, 8-Br-cGMP increased fictive swim frequency as recorded from a single *Clione* Pd-7 swim interneuron. The resting membrane potential and fictive frequency for each recording are: control resting membrane potential equals −59 mV and fictive swim frequency equals 2.19 s^−1^; 8-Br-cGMP (200 μM) resting membrane potential equals −57 mV and fictive swim frequency equals 3.66 s^−1^; wash: resting membrane potential equals −59 mV and fictive swim frequency equals 2.08 s^−1^. (**B**) Results showing a significant increase (65.0%) in mean fictive swim frequency averaged from 6 separate experiments involving the application of 200 μM 8-Br-cGMP. The mean fictive swim frequency increased significantly (the letters a and b indicate significant differences among means; letters that are the same are not significantly different) from 1.66 ± 0.150s^−1^ for the control to 2.74 ± 0.443s^−1^ for 200 μM 8-Br-cGMP (*N *=* *6; *P *=* *0.0055; Friedman test, nonparametric repeated measures ANOVA, with Dunn multiple comparison post-test).

Bath application of 200 µM 8-Br-cGMP, a cell membrane-permeable cGMP analog, revealed that cGMP produced swim acceleration in *Clione* (Fig. 1A shows the effect of 8-Br-cGMP on fictive swim frequency from one preparation). Figure 1B shows the mean fictive swim frequencies of control, 8-Br-cGMP, and wash activity from the 6 different animals used in the experiment. Following the application of 8-Br-cGMP, the mean fictive swim frequency increased significantly from 1.66 ± 0.150s^−1^ to 2.74 ± 0.443s^−1^ or 65% (*N *=* *6; *P *=* *0.0055; Friedman test, nonparametric repeated measures ANOVA, with Dunn multiple comparison post-test). The mean swim frequency after washing out 8-Br-cGMP in seawater (1.59 ± 0.120s^−1^) is not significantly different from the control mean swim frequency, indicating that the effect of 8-Br-cGMP is completely reversible.

In addition to 8-Br-cGMP, isoliquiritigenin (100 µM), a chemical that stimulates sGC to produce cGMP, increased mean fictive swim frequency—from 2.08 ± 0.335s^−1^ to 2.84 ± 0.319s^−1^ or 37%—however, this was not a significant difference (data not shown; *N *=* *5; unpaired *t*-test with Welch correction; *P *=* *0.1443; the data passed the Kolmogorov–Smirnov normality test).

**Fig. 2:**
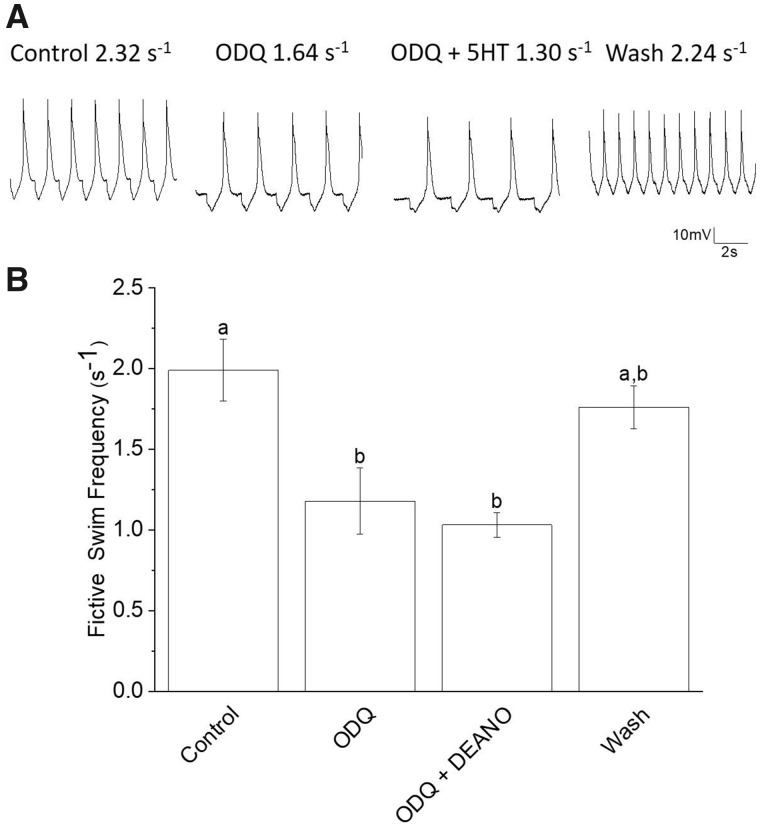
Effect of sGC inhibitor, ODQ (20 μM), on preventing NO-induced swim acceleration. ODQ applied for 15 min significantly slows the mean fictive swim frequency recorded from *Clione* Pd-7 swim interneurons. (**A**) An example of the application of 20 μM ODQ. In this example, ODQ decreased fictive swim frequency as recorded from a single *Clione* Pd-7 swim interneuron. The resting membrane potential and fictive frequency for each recording are: control resting membrane potential equals −53 mV and fictive swim frequency equals 2.32 s^−1^; ODQ (20 μM) resting membrane potential equals −62 mV and fictive swim frequency equals 1.64 s^−1^; ODQ (20 μM) plus the NO donor, DEANO (50 μM), resting membrane potential equals −63 mV and fictive swim frequency equals 1.30 s^−1^; wash resting membrane potential equals −56 mV and fictive swim frequency equals 2.24 s^−1^. (**B**) Results showing that co-application of the NO donor, DEANO (50 μM), following the 15 min of previously applied ODQ (20 μM) fails to significantly (the letters a and b indicate significant differences among means; letters that are the same are not significantly different) increase the fictive swim frequency (*N *=* *5; *P *=* *0.0006; Friedman test, nonparametric repeated measures ANOVA, with Dunn multiple comparison post-test). The wash is not significantly different from the either control or treatment groups.

The inhibitor of sGC, ODQ (20 µM), decreased fictive swim frequency and prevented NO, the typical endogenous activator of sGC, from inducing swim acceleration (Fig. 2). Application of 20 µM ODQ significantly decreased the mean fictive swim frequency from 1.99 s^−1^ to 1.18 s^−1^—a decrease of 41%. Additionally, the application of DEANO (50 µM) did not increase the mean fictive swim frequency in preparations previously treated with ODQ. The mean fictive swim frequency when 50 µM DEANO is added to preparations previously treated with ODQ is 1.03 s^−1^, which is a 48% decrease in mean fictive swim frequency compared to the control. The washing out combined ODQ and DEANO return the mean fictive swim frequency to 1.76 s^−1^, which is not significantly different than the mean fictive swim frequency of the control. Thus, the effects of co-application of ODQ and DEANO is completely reversible (*N *=* *5; *P *=* *0.0006; Friedman test, nonparametric repeated measures ANOVA, with Dunn multiple comparison post-test).

### The effect of cGMP on swim interneuron (Pd-7) action potential duration, membrane potential, and sag potential

**Fig. 3:**
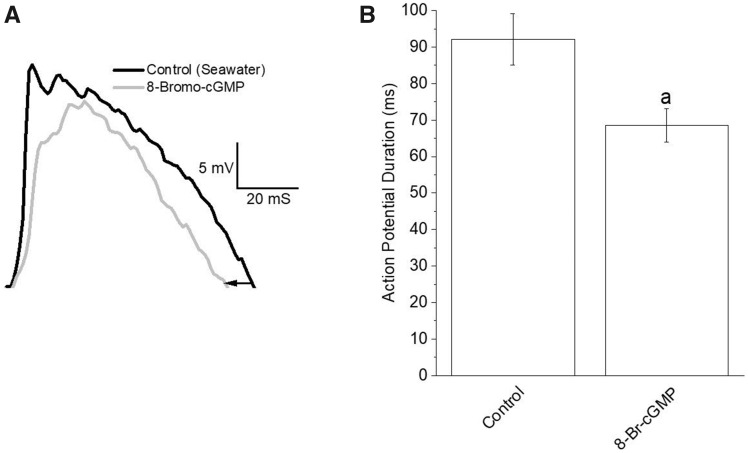
Reduced action potential duration (spike narrowing) in *Clione* Pd-7 swim interneuron produced by application of 200 μM 8-Br-cGMP. (**A**) Overlap of single Pd-7 action potentials in the absence (control; black trace) and presence of 200 μM 8-Br-cGMP (gray trace) showing spike narrowing (left-pointing arrow). (**B**) Results showing a significant (26%) decrease in the mean action potential duration averaged from 6 separate experiments involving the application of 200 μM 8-Br-cGMP. The mean action potential duration decreased from 92.1 ± 7.03 ms for the control to 68.5 ± 4.54 ms for 200 μM 8-Br-cGMP (*N *=* *6; *P *=* *0.0081; Friedman test, nonparametric repeated-measures ANOVA, with Dunn multiple comparison post-test).

The effect of cGMP on fictive swim frequency is accompanied by a simultaneous decrease in swim interneuron (Pd-7) action potential duration (i.e., spike narrowing). The effect of cGMP on spike duration is significant (*N *=* *6; *P *=* *0.0081; Friedman test, nonparametric repeated measures ANOVA, with Dunn multiple comparison post-test; Fig. 3). The mean action potential duration during the application of 200 µM 8-Br-cGMP is 68.5 ± 4.54 ms compared to 92.1 ± 7.03 ms in the absence of 200 µM 8-Br-cGMP (control)—thus, an 26% decrease in action potential duration occurs with the application of 8-Br-cGMP. The effect of 8-Br-cGMP on action potential duration is completely reversible as indicated by the wash in seawater (the mean action potential duration after washing out 8-Br-cGMP is 92.1 ms ± 8.26, which is not significantly different than the control).

**Fig. 4:**
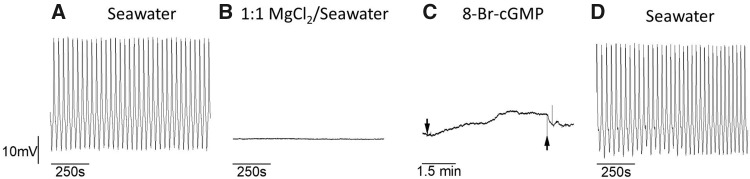
Change in resting membrane potential (baseline depolarization) in *Clione* Pd-7 swim interneuron produced by application of 200 μM 8-Br-cGMP in preparations bathed in a 1:1 mixture of isotonic (0.333 M) MgCl_2_ and filtered seawater. (**A**) Control fictive swim activity in filtered seawater. (**B**) Applying a mixture of 1:1 MgCl_2_/seawater inhibits fictive swim activity. This shows the effect of 1:1 MgCl_2_/seawater 3 min prior to adding 8-Br-cGMP. (**C**) Addition of 8-Br-cGMP to the preparation bathed in 1:1 MgCl_2_/seawater results in an 8.3 mV depolarization. The downward arrow indicates the time 8-Br-cGMP is added and the upward arrow indicates the beginning of the wash in filtered seawater. (**D**) The recovery of normal fictive swim activity following washing the preparation in filtered seawater.

In addition to spike narrowing, the application of 200 µM 8-Br-cGMP also results in baseline depolarization when applied to preparation bathed in a 1:1 mixture of seawater and isotonic (0.333 M) MgCl_2_ (*N *=* *3; Fig. 4). The resting membrane potential of Pd-7 interneurons in control conditions (seawater) is ∼−55.5 mV, whereas the mean resting membrane potential in these cells after application of 200 µM 8-Br-cGMP is ∼−47.2 mV—an ∼8.3 mV depolarizing shift in membrane potential. The sample size, *N *=* *3, is not sufficient for statistical comparison. However, this result is corroborated by findings previously reported in Moroz et al. 2000 that show depolarization in both general excitor (GE) motoneurons and swim interneurons exposed to DEANO and depolarization of GE when exposed to 8-Br-cGMP.

**Fig. 5:**
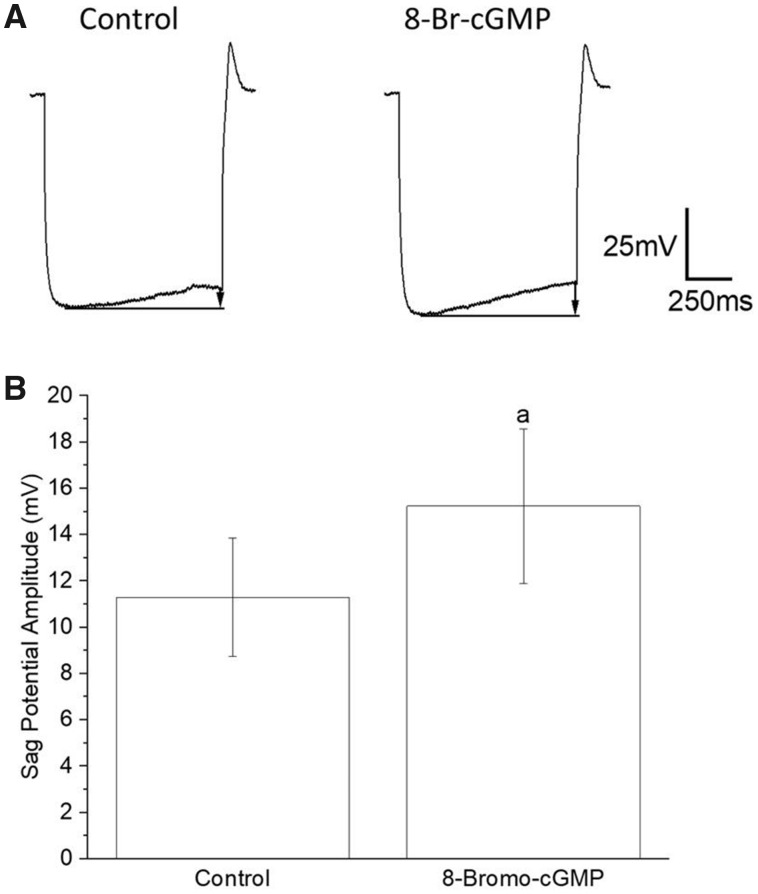
Change in sag potential in *Clione* Pd-7 swim interneuron produced by application of 200 μM 8-Br-cGMP in preparations chemically isolated by bath application of atropine (1 mM), TTX (1 μM), and CNQX (10 μM). (**A**) Enhanced sag potential amplitude produced by application of 200 μM 8-Br-cGMP as recorded from one *Clione* Pd-7 swim interneuron—control sag amplitude equals 6.54 mV and 8-Br-cGMP sag amplitude equals 11.27 mV (−1nA, 1 s duration current injection; resting potential for both control and 8-Br-cGMP recordings is −48 mV). (**B**) Results showing a significant (35%) change in the mean sag potential amplitude averaged from 5 separate experiments involving the application of 200 μM 8-Br-cGMP in preparations bathed in atropine (1 mM), TTX (1 μM), and CNQX (10 μM). The mean sag potential amplitude changed from 11.28 ± 2.55 mV for the control to 15.22 ± 3.34 mV for 200 μM 8-Br-cGMP (*N *=* *5; *P *=* *0.0354; paired *t*-test; the data passed the Kolmogorov–Smirnov assumption test with Kolmogorov–Smirnov distance = 0.27 and *P* > 0.05).

The effect of 200 µM 8-Br-cGMP on sag amplitude and PIR was tested using chemicals to synaptically isolate Pd-7 interneurons. Synaptic isolation involves the application of a combination of TTX (1 µM) to block action potential production, and the drugs CNQX (10 µM) and atropine (1 mM), antagonists that block glutamatergic and cholinergic receptors that mediate reciprocal inhibition between Pd-7 and Pd-8 swim interneurons (Panchin et al. 1995; Sadreyev and Panchin 2002; Pirtle and Satterlie 2004). Application of 200 µM 8-Br-cGMP to chemically isolated Pd-7 interneurons indicates that cGMP mediates a significant enhancement of the sag potential by 35%, from 11.28 ± 2.55 mV to 15.22 ± 3.34 mV (*N *=* *5; *P *=* *0.0354; paired two-tailed *t*-test; the data passed the Kolmogorov–Smirnov assumption test with Kolmogorov–Smirnov distance = 0.27 and *P* > 0.05; Fig. 5), which is consistent with the effect of 5HT on sag potential (Pirtle and Satterlie 2004, 2007).

### Effect of guanylyl cyclase inhibition and protein kinase G inhibition on 5HT-induced swim acceleration

**Fig. 6:**
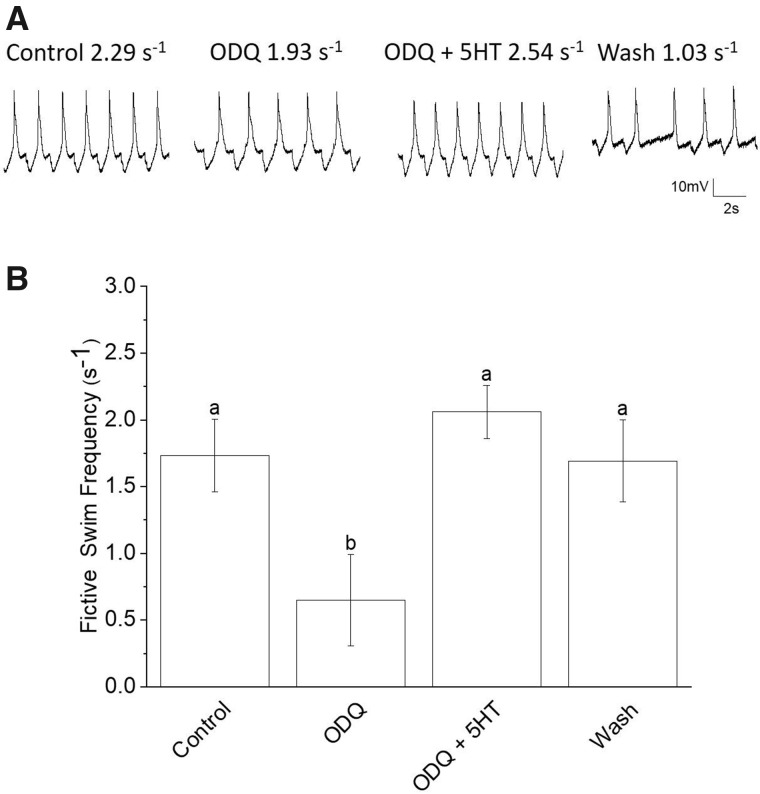
Application of the sGC inhibitor, ODQ (20 μM), does not inhibit 5HT-induced swim acceleration. (**A**) An example of the application of 20 μM ODQ. In this example, the application of ODQ for 15 min decreased fictive swim frequency as recorded from a single *Clione* Pd-7 swim interneuron. The resting membrane potential and fictive frequency for each recording are: control resting membrane potential equals −55 mV and fictive swim frequency equals 2.29 s^−1^; ODQ (20 μM) resting membrane potential equals −53 mV and fictive swim frequency equals 1.93 s^−1^; ODQ (20 μM) plus 5HT (1 μM) resting membrane potential equals −52 mV and fictive swim frequency equals 2.54 s^−1^; wash resting membrane potential equals −47 mV and fictive swim frequency equals 1.03 s^−1^. (**B**) ODQ applied for 15 min significantly slows the mean fictive swim frequency recorded from 6 separate *Clione* Pd-7 swim interneurons. Co-application of 5HT (1 μM), following the 15 min of ODQ (20 μM) exposure significantly (the letters a and b indicate significant differences among means; letters that are the same are not significantly different) increased the fictive swim frequency above that of the ODQ mean fictive swim frequency (the wash mean fictive swim frequency is not included in the analysis; *N *=* *6; *P *=* *0.0081; Friedman test nonparametric repeated-measures ANOVA with Dunn multiple comparison post-test). The positive shift in membrane potential observed in the recording during the wash period activity is most likely due to the stability of the recording rather than a pharmacological phenomenon. The interneuron somas range between 20 and 35 μm in diameter (Satterlie 1985) and the recording is affected by how tightly pinned the preparation remains during the experiment. Because of the size of the interneurons and changes in the stability in the preparation, small movements during the wash period can displace the intracellular recording electrode.

Because cGMP mediates swim acceleration and many of the accompanying 5HT-induced cellular changes that occur during swim acceleration are produced by cGMP, we hypothesize that 5HT’s effect is mediated, at least in part, by cGMP. To test the hypothesis we applied, in separate experiments, ODQ and Rp-8-Br-PET-cGMP (Rp-8-Br-PET-cGMP is a drug that inhibits cGMP-dependent protein kinase [PKG]). Application of ODQ significantly decreased fictive swim frequency by 60%, from 1.73 ± 0.272s^−1^ to 0.689 ± 0.337s^−1^. Addition of 5HT following treatment with ODQ significantly increased fictive swim frequency to 2.12 ± 0.239s^−1^ when compared to ODQ alone (*N *=* *6; *P *=* *0.0081; Friedman test, nonparametric repeated measures ANOVA, with Dunn multiple comparison post-test; Fig. 6; the wash, with *N *=* *3, was not included in the analysis).

**Fig. 7:**
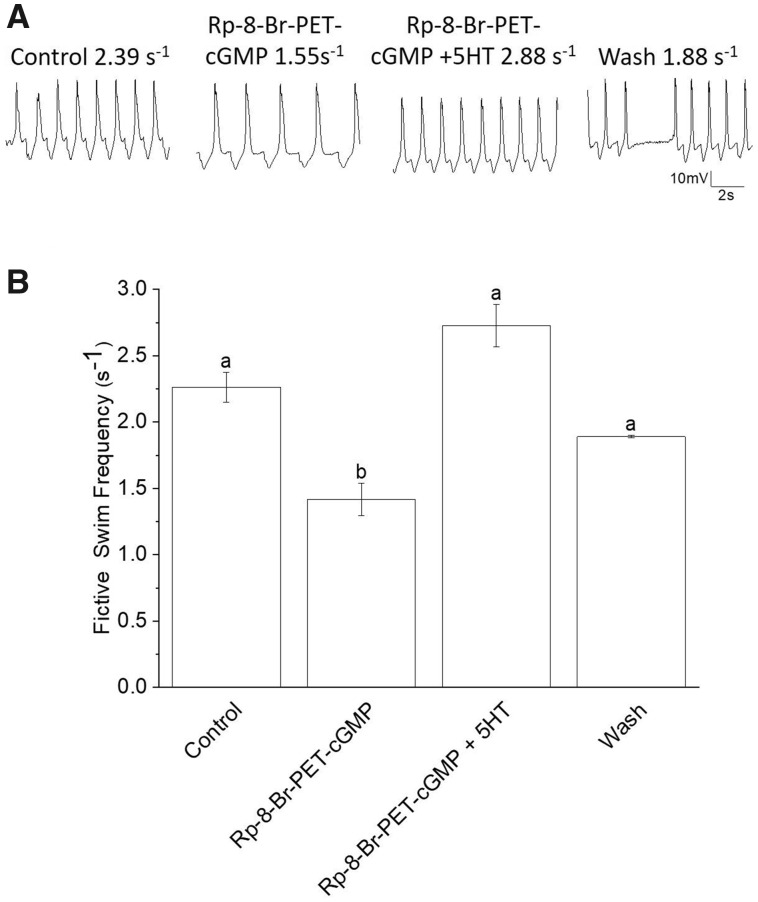
Application of the PKG inhibitor, Rp-8-Br-PET-cGMP (100 μM), does not inhibit 5HT-induced swim acceleration. (**A**) An example of the application of 100 μM Rp-8-Br-PET-cGMP. In this example, the application of Rp-8-Br-PET-cGMP for 15 min decreased fictive swim frequency as recorded from a single *Clione* Pd-7 swim interneuron. The resting membrane potential and fictive frequency for each recording are: control resting membrane potential equals −50 mV and fictive swim frequency equals 2.39 s^−1^; Rp-8-Br-PET-cGMP (100 μM) resting membrane potential equals −59 mV and fictive swim frequency equals 1.55 s^−1^; Rp-8-Br-PET-cGMP (100 μM) plus 5HT (1 μM) resting membrane potential equals −64 mV and fictive swim frequency equals 2.88 s^−1^; wash resting membrane potential equals −69 mV and fictive swim frequency equals 1.88 s^−1^. (**B**) Rp-8-Br-PET-cGMP applied for 15 min significantly slows the mean fictive swim frequency recorded from 6 separate *Clione* Pd-7 swim interneurons. Co-application of 5HT (1 μM), following the 15 min of Rp-8-Br-PET-cGMP exposure significantly (the letters a and b indicate significant differences among means; letters that are the same are not significantly different) increased the fictive swim frequency (the wash mean fictive swim frequency is not included in the analysis; *N *=* *6; *P *=* *0.0001.Friedman test nonparametric repeated-measures ANOVA with Dunn multiple comparison post-test). The positive shift in membrane potential observed in the recording during the wash period activity is most likely due to the stability of the recording rather than a pharmacological phenomenon. The interneuron somas range between 20 and 35 μm in diameter (Satterlie 1985) and the recording is affected by how tightly pinned the preparation remains during the experiment. Because of the size of the interneurons and changes in the stability in the preparation, small movements during the wash period can displace the intracellular recording electrode.

Application of Rp-8-Br-PET-cGMP decreased the mean fictive swim frequency by 33%, from 2.13 ± 0.112s^−1^ to 1.42 ± 0.122s^−1^. However, this decrease in mean fictive swim frequency is not significant. The addition of 5HT following treatment with Rp-8-Br-PET-cGMP increased the mean fictive swim frequency to 2.73 ± 0.159s^−1^, which is significantly greater than the mean fictive swim frequency in Rp-8-Br-PET-cGMP alone. Hence, 5HT significantly increased the mean swim frequency, by 92%, when protein kinase G is inhibited (*N *=* *6; *P *=* *0.0001; Friedman test, nonparametric repeated measures ANOVA, with Dunn multiple comparison post-test; Fig. 7; the wash, with *N *=* *2, was not included in the analysis).

**Fig 8.**
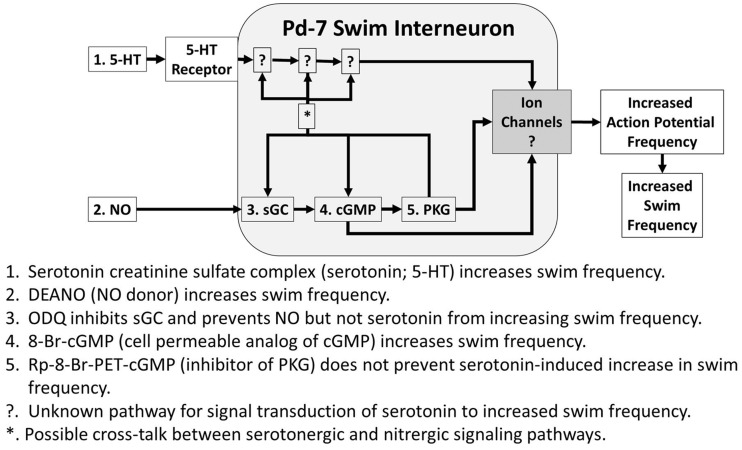
Both 5HT creatinine complex (1; 5HT) and the NO donor DEANO (2; NO) increased the fictive locomotor frequency recorded from Pd-7 swim interneurons that comprise the *Clione* locomotor CPG and control the dorsal movement of the parapodia during swimming. Each action potential of a Pd-7 swim interneuron synaptically produces a single burst of action potentials in synergistic motoneurons, and each burst of action potentials produced by the motoneuron contributes to the contraction of synergistic muscle fibers in the parapodia. Thus, the increase in action potential frequency in Pd-7 swim interneurons directly correlates to the frequency of parapodial movements. Application of the cGMP analog, 8-Br-cGMP (4) increased swim frequency. Hence, we hypothesize that 5HT and NO may produce their effects on Pd-7 swim interneurons through a common signaling pathway mediated by cGMP or alternatively through separate, but parallel pathways that converge to produce a common result. Our data suggest the later hypothesized mechanism. ODQ (3), an inhibitor of sGC, the enzyme that synthesizes cGMP from GTP, when applied inhibited NO-induced but not 5HT-induced swim acceleration (these results are shown in Figs. 2 and 6). Similarly, 5HT is capable of inducing swim acceleration when the PKG inhibitor, Rp-8-Br-PET-cGMP (5) is applied, indicating that 5HT contributes to swim acceleration without the activation of PKG. It is unknown what signaling mechanism mediates 5HT-induced swim acceleration (?), and experiments are planned to elucidate these details. Because the effect of 5HT is blocked by the 5HT antagonist, mianserin, the most likely serotonin receptor involved is the 5HT2-like receptor, a metabotropic receptor that is linked to phospholipase C signal transduction mechanisms (Barbas et al. 2003). While our results show that 5HT is capable of inducing swim acceleration independently of cGMP there remains the possibility of cross-talk between the serotonergic and nitrergic signaling (Nishizuka 1992; Selbie and Hill 1998).

## Discussion

Heterobranch (formerly classified as Opisthobranch) mollusks (Schrödl et al. 2011; Kocot et al. 2013) are principally bottom-dwelling creatures and thus locomote by means of muscular contraction or ciliary beating associated with the foot (Katz et al. 2001; Willows 2001; Chase 2002). Swimming locomotion in Heterobranchs is an evolutionary adaptation that requires the foot or structural modifications of the foot (e.g., parapodia as observed in *C. limacina*), and is propelled by dorsal-ventral body flexions, lateral body flexions, writhing movements of the foot, or flapping of parapodia (Willows 2001). Furthermore, Willows’ (2001) comparison of swimming locomotion among several Heterobranch species suggests that swimming locomotion may be a beneficial adaptation among these species that allows for food acquisition, “reproductive dispersal,” and predator avoidance (Mills 1994; Willows 2001). Katz et al. (2001) propose that the influence of serotonergic modulation of swimming in Heterobranch mollusks arose from an ancestral serotonergic arousal system and that the serotonergic neurons that modulate swimming in Heterobranchs exhibit homology (Katz et al. 2001; Newcomb and Katz 2009).

Homologous serotonergic neurons among evolutionarily allied molluscan species may mediate different behavioral dynamics (Katz et al. 2001; Newcomb and Katz 2009). This is true for serotonergic neurons that modulate swimming locomotor behavior in *Clione*, *Melibe*, *Pleurobranchaea*, and other Heterobranch mollusks (Katz et al. 2001; Newcomb and Katz 2007). In *Clione*, *Melibe*, and *Pleurobranchaea*, these homologous serotonergic neurons are the Cr-SP neurons, CeSP-A neurons, and As1-3 neurons, respectively (Newcomb and Katz 2007). The serotonergic homologs of *Clione* (Cr-SP neurons), *Melibe* (CeSP-A neurons), and *Pleurobranchaea* (As1-3 neurons) are located similarly in the dorsal posterior aspect of the cerebral ganglia (Satterlie et al. 1995; Sudlow et al. 1998; Jing and Gillette 1999; Newcomb et al. 2006). As an example of this structural and functional homology, depolarization of both Cr-SP and CeSP-A neurons during episodes of inactivity initiate and sustain swimming in both *Clione* and *Melibe*. Furthermore, depolarization of both Cr-SP and CeSP-A excites both *Clione* and *Melibe* swim interneurons (Satterlie and Norekian 1995; Newcomb and Katz 2009). Thus, many aspects of 5HT on locomotor behavior are similar across distantly related Heterobranch mollusks.

While 5HT is similar among related molluscan species, NO is different—at least when comparing the pharmacological effects of NO on swimming locomotor behaviors among different Heterobranch mollusks using NO donors, inhibitors of NOS, and chemicals that inactivate NO (Moroz and Gillette 1995, 1996; Moroz et al. 2000; Newcomb and Watson 2001, 2002). However, there is a paucity of data on the electrophysiological properties and synaptic connectivity of these nitrergic neurons to swim interneurons and swim motoneurons in these animals. Much of what we know of the role of NO in mollusks focuses on feeding behaviors (Elphick et al. 1995; Moroz and Gillette 1995; 1996; Huang et al. 1998; Hatcher et al. 2000, 2006; Lovell et al. 2000). Moroz and Gillette (1995, 1996), propose that there are dietary differences in nitrergic modulatory systems among the Mollusca based on whether the species is carnivorous or herbivorous.

Our results here and in previously reported experiments both demonstrate that 5HT and NO increase locomotor speed in *C. limacina* (Satterlie and Norekian 1995; Panchin et al. 1996; Satterlie and Norekian 1996; Moroz et al. 2000). Here we provide evidence that 5HT and NO produce their effects independently of each other—selectively inhibiting either serotoninergic or nitrergic system does not preclude the uninhibited system from producing swim acceleration in *Clione*. Thus, in *Clione*, there are 2 separate modulatory systems—a serotonergic system and a nitrergic system—that contribute to locomotor acceleration. We hypothesize that 5HT and NO converge onto a common cGMP pathway to modulate swimming locomotion in *Clione*. However, our data indicate that cGMP mediates NO-induced changes in *Clione* swimming speed but does not completely account for 5HT-induced changes in *Clione* swimming speed.

Application of 200 µM of the membrane-permeable analog of cGMP, 8-Br-cGMP, significantly increased the fictive locomotor frequency by 65% as recorded from Pd-7 swim interneurons. Additionally, while not significant, there was an increase in fictive locomotor speed with the application of isoliquiritigenin (100 µM), an activator of sGC. Blocking sGC with ODQ significantly reduced swim locomotor frequency in *Clione*, thus suggesting that basal levels of sGC activity play a role in maintaining slow swimming. ODQ also prevented NO-induced swim acceleration, indicating NO stimulates sGC and that the subsequent production of cGMP is an important second messenger mediating the NO-induced swim acceleration. A volume of 20 mL of filtered seawater was perfused through the recording dish to wash out the effects of drugs. However, because fictive swim frequency recorded during the washing activity is not significantly different than that the fictive swim frequency recorded in the presence of ODQ or ODQ+DEANO, there may be some residual effects of ODQ influencing our wash activity.

In addition to increasing the fictive swim frequency, 8-Br-cGMP had several noted effects on Pd-7 swim interneurons. These effects include a significant (26%) decrease in action potential duration (i.e., spike narrowing), a positive shift in resting membrane potential (i.e., baseline depolarization), and a significant (35%) depolarized shift in the sag potential (Figs. 3–5). All of these effects of 8-Br-cGMP are consistent with the effect that 5HT has on the *Clione* swim locomotor rhythm (Satterlie 1991; Satterlie et al. 2000), and indicate a role of cGMP in mediating the serotonergic modulation of swimming locomotion in *Clione*. To test our hypothesis, we co-applied 5HT with previously applied chemicals that block 2 key steps in the cGMP signal transduction pathway—ODQ, which blocks sGC production of cGMP and Rp-8-PET-cGMP, which blocks PKG.

When 5HT is co-applied to preparations previously treated with ODQ the fictive swim frequency significantly increased 208%. When 5HT is co-applied to preparations previously treated with Rp-8-Br-PET-cGMP the fictive swim frequency significantly increased by 92%. These values of fictive swim frequency are significantly greater than the values of fictive swim frequencies recorded in ODQ or Rp-8-Br-PET prior to the application of 5HT. Thus, 5HT is capable of increasing swim speed independent of cGMP. Therefore, a different signaling pathway must be contributing to the effect that 5HT has on the *Clione* swim locomotor rhythm.

A noteworthy observation regarding our data is the difference between the mean fictive swim frequencies recorded with ODQ and Rp-8-Br-PET-cGMP. This difference may be explained because of the role that cyclic nucleotides have on hyperpolarization-cyclic nucleotide-gated (HCN) ion channels—cGMP affects HCN channels directly by binding to the cyclic nucleotide-binding domain of HCN channels or indirectly through activation of a PKG and subsequent phosphorylation of HCN channels (Biel et al. 2002). We have shown previously that block of HCN ion channels by ZD7288 reduces fictive swim frequency, reduces sag potential amplitude, and inhibits 5HT-induced acceleration in *Clione* (Pirtle et al. 2010). While both ODQ and Rp-8-Br-PET-cGMP decreased fictive swim frequency, the effect of ODQ was greater than that of Rp-8-Br-PET-cGMP—ODQ decreased fictive swim frequency 41–60% (from separate experiments in which ODQ was applied) while Rp-8-Br-PET-cGMP decreased fictive swim frequency 33% (Figs. 6 and 7). ODQ inhibits sGC, thus preventing the production of cGMP. In this situation, less PKG would be activated because of less cGMP production following treatment with ODQ. In contrast, Rp-8-Br-PET-cGMP inhibits PKG without precluding the production of cGMP. Consequently, ODQ would have a greater effect on the *Clione* swim locomotor rhythm than Rp-8-Br-PET-cGMP because ODQ would directly prevent the production of cGMP and indirectly reduce activation of PKG.

Our results raise an important question—what other signaling pathways mediate the effect of 5HT-induced acceleration on the *Clione* swim locomotor rhythm? One possibility is that 5HT has its effect on the *Clione* swim CPG through a cAMP pathway. Our preliminary results indicate that rather than inducing swim acceleration in *Clione*, cAMP slows swimming locomotor speed. Calcium has an effect on PIR of *Clione* swim interneurons (Pirtle and Satterlie 2007), and one possibility that we are currently exploring is the role of a calcium signaling pathway in modifying *Clione* swim activity. 5HT can initiate signal transduction through phospholipase in mollusks (Barbas et al. 2003), and therefore might activate a phospholipase C-DAG/IP3–calcium–calmodulin pathway to increase fictive swim frequency in *Clione*. The role of calcium signaling through phospholipase C, IP3, DAG, increased intracellular Ca^++^, and calmodulin is currently being investigated. Furthermore, this mechanism may play an important role in enhancing the synaptic efficacy of *Clione* swim CPG interneurons during swim acceleration. *Clione* swim interneurons exhibit spike narrowing (decreased action potential duration) as a necessary consequence of increased swim cycle frequency during acceleration (Satterlie et al. 2000). This necessary change in action potential duration, however, counteracts the effectiveness of swim interneuron synaptic communication (Satterlie and Norekian 2001). A calcium signal transduction mechanism enhances synaptic efficacy in hippocampal pyramidal cells (Bramham, et al. 1994) and this mechanism may participate to enhance synaptic efficacy during *Clione* swim acceleration as a countermeasure to the effects of spike narrowing.

The modulatory effects of 5HT and NO on the *Clione* swim CPG shares some similarities and differences with serotonergic and nitrergic modulation of swim locomotor CPGs of other Heterobranch species. For example, in *Melibe*, Newcomb and Watson (2002) and Lewis et al. (2011) have shown, respectively, that swimming locomotion in *Melibe* is inhibited by NO and cGMP but stimulated by 5HT (5HT initiates swimming and controls swimming speed in *Melibe*). This contrasts greatly with *Clione* swimming locomotion, where both NO and 5HT are excitatory as shown in our data here and previously (Panchin et al. 1996; Moroz et al. 2000; Satterlie et al. 2000; Satterlie and Norekian 2001; Pirtle et al. 2010). Similarly, aspects of serotonergic and nitrergic modulation can be compared between *Clione* and *Pleurobranchaea.* 5HT excites swim CPG neurons in *Clione* and *Pleurobranchaea* (Jing and Gillette 2000). While evidence suggests that NO excites aspects of feeding behavior in both *Clione* and *Pleurobranchaea* (Moroz and Gillette 1996; Hurst et al. 1999; Hatcher et al. 2000, 2006; Moroz et al. 2000), there is currently little research to compare the effects of NO on swimming locomotor behavior between these 2 species.

Swimming locomotion is characteristically distinct in *Clione*, *Melibe*, and *Pleurobranchaea* and relates to each species’ ecological niche as it pertains to feeding, escape from predators, and reproduction (Willows 2001). Unlike *Melibe* and *Pleurobranchaea*, *Clione* are classified as holoplanktonic animals and are therefore almost continuously swimming to maintain their position within the water column by means of dorsal–ventral movements of its wing-like parapodia. Additionally, *Clione* are predatory specialists that actively hunt their prey, *Limacina helicina* (Conover and Lalli 1972; Litvinova and Orlovsky 1985; Lalli and Gilmer 1989; Hermans and Satterlie 1992; Norekian1995). The principal mode of locomotion in *Melibe* and *Pleurobranchaea* is crawling. Swimming locomotion in *Melibe* involves lateral flexion of the animal’s whole body and occurs when displaced from its typical attachment to eelgrass and may also be initiated to avoid predators such as the sun star, *Pycnopodia sp*. (Watson, et al. 2001). Additionally, swimming in *Melibe* is related to the animal’s reproductive dispersal and seasonal differences in food availability (Mills 1994). Swimming locomotion in *Pleurobranchaea* involves dorsal-ventral flexion of the animal’s whole body and is an escape response (Jing and Gillette 1995, 1999)—typically as a mechanism for predatory avoidance (Gillette and Jing 2001).

The differences in swimming mode and feeding strategy among *Clione*, *Melibe*, and *Pleurobranchaea* may explain differences in the modulatory effects of both 5HT and NO in these species. *Clione* are active predators that require fine-tuned adjustments in their locomotor behavior during hunting and prey capture. Moreover, swimming acceleration occurs during hunting and feeding in *Clione* (Conover and Lalli 1972; Litvinova and Orlovsky 1985; Lalli and Gilmer 1989; Hermans and Satterlie 1992; Norekian1995). Therefore, because swimming, hunting behavior, and feeding occur together in *Clione*, it is tenable that serotonergic and nitrergic systems merged as an adaptation to control *Clione* locomotion. This contrasts with both *Melibe* and *Pleurobranchaea* where feeding behavior and swimming locomotor behavior are mutually exclusive. *Melibe* are suspension feeders that acquire their prey by opening and closing an extended oral hood (Watson and Trimarchi 1992). Newcomb and Watson (2002) suggest that feeding behavior and swimming locomotion in *Melibe* are “mutually exclusive” behaviors—feeding behavior inhibits swimming behavior. Newcomb and Watson (2002) further suggest that the inhibitory effect of *Melibe* feeding behavior on swimming behavior may be mediated by NO. Swim inhibition during feeding makes sense given that the suspension-feeding strategy of *Melibe* would be much more efficient if the animal crawls while attached to a substrate (i.e., eel grass). Similarly, in *Pleurobranchaea*, crawling is the principal mode of locomotion, and activation of swimming is a means to avoid predation. However, unlike *Clione* where swimming and feeding behaviors occur together, swimming and feeding behavior in *Pleurobranchaea* are mutually exclusive behaviors—escape from predators takes priority over feeding when it comes to *Pleurobranchaea* survival (Jing and Gillette, 1995, 1999).

Our data show that 5HT and NO accelerate swimming locomotor behavior in *Clione*, and that these 2 neuromodulatory systems may operate independently of the other. Furthermore, our data show that the effect of NO on *Clione* swim acceleration is dependent on cGMP. 5HT on the other hand does not produce acceleration in *Clione* via an exclusive cGMP-dependent pathway. Preliminary data have ruled out that 5HT functions through cAMP as these experiments indicate that cAMP inhibits swimming locomotion in *Clione*. We are currently exploring the possibility that 5HT functions via a phospholipase C-protein kinase C-calcium signal transduction mechanism. The interactions of 5HT and NO signaling in *Clione* represent an emerging area of complexity in the control of the central pattern generation of locomotor behavior (Fig. 8). In addition to neuronal networks, synaptic, and cellular properties in controlling aspects of central pattern generation of locomotion are biochemical-signaling networks. *Clione* has and will continue to serve as a tractable system to further our understanding of how signal transduction mechanisms interact to produce variability in locomotor CPGs.

## Funding

This publication was made possible by an Institutional Development Award (IDeA) from the National Institute of General Medical Sciences of the National Institutes of Health under Grant #P20GM103408 to T.J.P. and by the M.J. Murdock Charitable Trust (Grant No. 2013175: MNL: 11/21/2013 to T.J.P.) (http://www.murdock-trust.org).

## Conflict of interest

None.
